# Genomic profile of *MYCN* non-amplified neuroblastoma and potential for immunotherapeutic strategies in neuroblastoma

**DOI:** 10.1186/s12920-020-00819-5

**Published:** 2020-11-10

**Authors:** Eunjin Lee, Ji Won Lee, Boram Lee, Kyunghee Park, Joonho Shim, Keon Hee Yoo, Hong Hoe Koo, Ki Woong Sung, Woong-Yang Park

**Affiliations:** 1 Samsung Genome Institute, Samsung Medical Center, 81 Irwon-ro, Gangnam-gu, Seoul, 06351 Republic of Korea; 2 Department of Pediatrics, Samsung Medical Center, Sungkyunkwan University School of Medicine, 81 Irwon-ro, Gangnam-gu, Seoul, 06351 Republic of Korea; 3 Department of Health Science and Technology, Samsung Advanced Institute for Health Sciences and Technology, Sungkyunkwan University, Seoul, Korea; 4 Department of Molecular Cell Biology, Sungkyunkwan University School of Medicine, Suwon, Korea

**Keywords:** *MYCN* non-amplified neuroblastoma, Tumour mutation burden, Mutational signature, Genomic profile, Immunotherapy

## Abstract

**Background:**

*MYCN* amplification is the most important genomic feature in neuroblastoma (NB). However, limited studies have been conducted on the *MYCN* non-amplified NB including low- and intermediate-risk NB. Here, the genomic characteristics of *MYCN* non-amplified NB were studied to allow for the identification of biomarkers for molecular stratification.

**Methods:**

Fifty-eight whole exome sequencing (WES) and forty-eight whole transcriptome sequencing (WTS) samples of *MYCN* non-amplified NB were analysed. Forty-one patients harboured WES and WTS pairs*.*

**Results:**

In the *MYCN* non-amplified NB WES data, maximum recurrent mutations were found in *MUC4* (26%), followed by *RBMXL3* (19%), *ALB* (17%), and *MUC16* and *SEPD8* (14% each). Two gene fusions, *CCDC32*-*CBX3* (10%) and *SAMD5*-*SASH1* (6%), were recurrent in WTS analysis, and these fusions were detected mostly in non-high-risk patients with ganglioneuroblastoma histology. Analysis of risk-group-specific biomarkers showed that several genes and gene sets were differentially expressed between the risk groups, and some immune-related pathways tended to be activated in the high-risk group. Mutational signatures 6 and 18, which represent DNA mismatch repair associated mutations, were commonly detected in 60% of the patients. In the tumour mutation burden (TMB) analysis, four patients showed high TMB (> 3 mutations/Mb), and had mutations in genes related to either MMR or homologous recombination. Excluding four outlier samples with TMB > 3 Mb, high-risk patients had significantly higher levels of TMB compared with the non-high-risk patients.

**Conclusions:**

This study provides novel insights into the genomic background of *MYCN* non-amplified NB. Activation of immune-related pathways in the high-risk group and the results of TMB and mutational signature analyses collectively suggest the need for further investigation to discover potential immunotherapeutic strategies for NB.

## Background

Neuroblastoma (NB), the most common extracranial solid tumour in children, accounts for 6 to 10% of all childhood cancers. NB arises from precursor cells of the sympathetic nervous system and adrenal medulla [1]. The clinical course is highly heterogeneous, ranging from spontaneous regression without therapeutic intervention to rapid progression to death, despite modern intensive multimodal treatment regimens. Thus, clinical and biological factor-based risk stratification and tailored treatment approaches have been the mainstay of NB treatment. International Neuroblastoma Risk Group (INRG) defines the high-risk group to include patients with *MYCN* amplified tumours and patients > 18 months old with metastatic tumours [2].

Amplification of the *MYCN* oncogene is the first genetic marker reported to indicate highly aggressive and advanced-stage NB. It is observed in approximately 20% of cases and remains a powerful prognostic factor, indicating adverse clinical outcomes [3]. The clinical features of *MYCN*-amplified NB have been attributed to the biological consequence of *MYCN* amplification. *MYCN*-amplified tumours make up about 40% of high-risk NBs [4], indicating that 60% of high-risk NBs are *MYCN* non-amplified tumours. Despite the extensive study of the genomic characteristics of high-risk NB including *MYCN*-amplified tumours [4–6], genomic profiling of *MYCN* non-amplified NB, including low- and intermediate-risk NB, has been limited.

Immunotherapy, which includes the use of immune checkpoint inhibitors, has become a potential therapeutic option, especially in adult oncology, and tumour mutational burden (TMB) is known to be a predictive marker for immunotherapy in many studies [7, 8]. However, except for a monoclonal antibody that acts against the tumour-associated disialoganglioside, GD2 [9], little is known about immunotherapy in NB. Here, we examined the genomic profiles of *MYCN* non-amplified NB and studied risk-group-specific biomarkers, TMB, and mutational signature to identify biomarkers for the molecular stratification of NB.

## Methods

### Study population and data collection

From November 2015, tissue and blood samples were collected prospectively from NB patients undergoing biopsy. Samples from patients who were diagnosed before November 2015 that had been deposited at the Samsung Medical Center Bio Bank were also included. Medical records regarding age, sex, stage, risk group, pathology, and outcome were collected. Tumour staging was determined by following the International Neuroblastoma Staging System standards [2]. *MYCN* amplification was determined by performing interphase fluorescence in situ hybridization on tumour tissues. Patients older than 18 months and in stage four malignancy and patients with *MYCN*-amplified tumours were stratified as high-risk patients.

### DNA and RNA extraction

All tumour specimens were reviewed by a pathologist to determine the percentage of viable tumours and their adequacy for sequencing. Genomic DNA from the tissue and blood was extracted using a QIAamp DNA Mini Kit (Qiagen, Valencia, CA, USA). The total RNA from the same fresh frozen tumour tissues was extracted with an RNeasy Mini Kit (Qiagen, Valencia, CA, USA), according to the manufacturer’s instructions. The quality and quantity of extracted nucleic acids were evaluated using Nanodrop 8000 UV–Vis spectrometer (NanoDrop Technologies Inc., Wilmington, DE, USA), Qubit ® 3.0 Fluorometer (Life technologies Inc., Carlsbad, CA, USA) and 4200 TapeStation (Agilent Technologies Inc., Santa Clara, CA, USA). Specimens with a yield over 100 ng were selected for whole exome sequencing (WES) and whole transcriptome sequencing (WTS). Those with a median DNA fragment size of 350 bp and an RNA integrity number (RIN) of 5 were selected.

### WES and variant calling

Tumour and matched normal DNA were enriched for exon regions, using the SureSelect XT regent kit (Agilent Technologies Inc., Santa Clara, CA, USA) and SureSelect XT Human All Exon V5 kit (Agilent Technologies Inc., Santa Clara, CA, USA). The libraries were pooled, denatured, and sequenced in 100-bp paired-end mode using the HiSeq Rapid SBS Kit v2 (200 Cycles) and HiSeq® Rapid PE Cluster Kit v2 in Illumina HiSeq 2500 platforms (Illumina Technologies Inc., San Diego, CA, USA). The mean target coverages were 166 × in tumours and 104 × in normal blood. Reads were aligned to the human reference genome (hg19) using the Burrows-Wheeler Alignment tool (BWA) version 0.7.5a [10]. Sequence Alignment and Mapping (SAM) files were converted to Binary Alignment and Mapping (BAM) files using SAMtools (v0.1.19) [11]. Duplicate reads were removed using Picard (version 1.128), base quality was recalibrated, and local realignment was optimized using The Genome Analysis Toolkit (GATK) version 3.5 [12]. Single nucleotide variants (SNVs) and indels were identified using MuTect2 version 3.8.0 [13], Strelka2 version 2.8.2 [14], and Pindel version 0.2.5b9 [15]. Germline variants were identified using HaplotypeCaller version 3.8.0 [16]. Variants were annotated using Ensembl Variant Effect Predictor (VEP) version 87 [17]. Variants located in exons with sufficient coverage (minimum depth of coverage ≥ 8) and a significant variant allele frequency (VAF ≥ 1%) were chosen for further statistical analyses. Synonymous variants were filtered out. Read alignments were manually examined using Integrative Genomic Viewer (IGV) (https://www.broadinstitute.org/igv/).

### WTS and data processing

Sequencing libraries were prepared using TruSeq RNA Sample Preparation kit v2 (Illumina Technologies Inc., San Diego, CA, USA). RNA libraries were sequenced in 100-bp paired-end mode using TruSeq Rapid PE Cluster kit and TruSeq Rapid SBS kit v2 in Illumina HiSeq 2500 (Illumina Technologies Inc., San Diego, CA, USA). Unresolved bases in FASTQ files were trimmed, reads were aligned to the human reference genome, hg19, using TopHat version 2.0.6 [18], and reference-guided assembly of transcripts was performed using Cufflinks version 2.1.1 [19]. Alignment quality was verified with SAMtools version 0.1.19 [11]. Gene expression was estimated from the RNASeq data of 56 patients using a count-based method with RSEM [20]. In total, 20,345 protein-coding genes were selected. Further, genes that were expressed in at least three samples were retained. A total of 16,120 genes were analysed. Gene counts were used as the input for Trimmed Mean of M value (TMM) normalization in the R package, edgeR [21], and normalized counts were transformed to log2-counts per million (logCPM) using the voom application in the R package, limma [22].

Gene fusions were predicted by several algorithms, such as ChimeraScan [23], deFuse [24], FusionMap [25], MapSplice [26], and TopHat [18]. Fusions predicted by more than three algorithms were considered further. The putative fusions were manually investigated using IGV.

### Validation of fusions by RT-PCR and Sanger sequencing

The putative gene fusions, detected by RNA-Seq, were verified by reverse transcription PCR (RT-PCR), followed by Sanger sequencing. cDNA was synthesized from 2 µg total RNA using a QuantiTect Reverse Transcription kit (Qiagen Inc., Hilden, Germany) with primers that flank the breakpoint of the fusion, in DNA Engine Tetrad 2 Peltier Thermal Cycler (BIO-RAD, Hercules, CA, USA) with the following cycling conditions: one cycle of 5 min at 95 °C, followed by three-step cycles of 30 s at 95 °C, 30 s at 62 °C, 10 min at 72 °C, and a final extension for 20 min at 72 °C. PCR products were purified using a Multiscreen filter plate (Millipore Corp., Bedford, MA, USA) and sequenced in an ABI prism 3730XL Analyzer (Thermo Fisher Scientific, Waltham, MA, USA) using a BigDye (R) Terminator v3.1 Cycle Sequencing Kit (Applied Biosystems Inc., Foster City, CA, USA). The results were accessed by Variant Reporter Software v 1.1 (Applied Biosystems Inc., Foster City, CA, USA).

### Mutational signatures and tumour mutation burden (TMB)

A set of 30 mutational signatures, which represent distinct characteristics of human cancer types based on base substitutions at the site of mutation, was obtained [27]. To calculate mutational signatures from each sample, deconstructSigs (R package) was used, and the weighted combination of predefined signatures was identified to comprehend the mutational profiles [28].

TMB, defined as the number of somatic variants per megabase (Mb), was calculated by dividing the total number of mutations from WES by the size of the target coding region.

### Gene-set enrichment analyses (GSEA)

Gene-set enrichment analyses (GSEA), based on gene expression data for each sample, were performed using R package, GSVA [29] on 17,810 annotated gene sets from the Molecular Signatures Database (MSigDB v6.2, https://software.broadinstitute.org/gsea/msigdb/index.jsp).

### Statistical analyses

All statistical tests were performed using R software v.3.4.2 (https://www.r-project.org/). The associations between risk-group and genomic information, including the frequency of mutation, TMB, mutational signature, gene expression, and gene-set expression, were examined using the T-test or Fisher’s exact test. Multiple test correction with false discovery rate (FDR) was applied to the expression data analyses. *P* value < 0.05 was considered as significant.

## Results

### Characteristics of patients

**Fig. 1:**
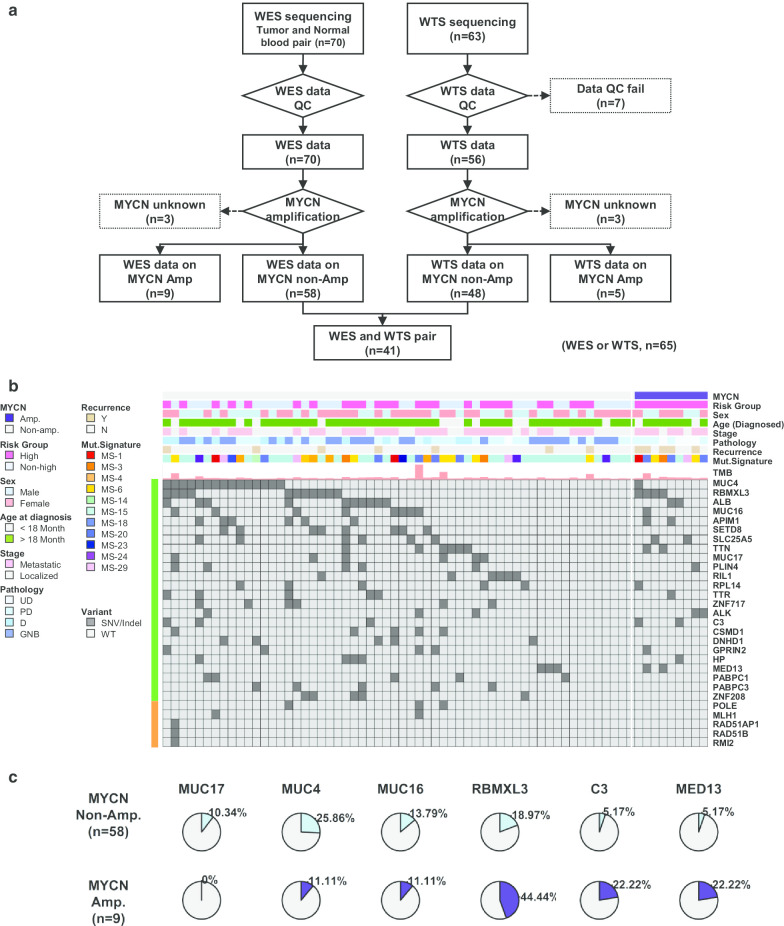
**a** Consort flow of study population. **b** Somatic mutation profiles of WES data. *MYCN*-amplified patients (n = 9), presented on the right side of the heatmap (purple), were compared with the non-amplified population. The top 20 frequently mutated genes (green bar) and 5 functional genes (orange bar) related to DNA mismatch repair or homologous recombination repair are listed. *TMB: Tumour Mutational Burden. **c** Comparison of incidence of mutation between *MYCN*-amplified and non-amplified groups

WES and WTS were conducted for 70 and 63 NB samples, respectively. QC filtering and removal of *MYCN-*amplified NB patient data yielded 58 WES and 48 WTS samples (Fig. 1a). All patients in this study were East Asian. Thirty-five patients (53.8%) were diagnosed as metastatic, and 26 patients (40%) were classified into the high-risk group. The median age was 3.1 years, with a range of 0–14.9 years. Seven patients (10.8%) experienced recurrence at median 1.7 (0.2–3.8) years (Table 1).

**Table 1:** Demographics of study populations for WES and WTS analysis

	All (n = 65)	WES (n = 58)	WTS (n = 48)
Sex, no. (%)			
Male	30 (46.2)	25 (43.1)	25 (52.1)
Female	35 (53.8)	33 (56.9)	23 (47.9)
Age, median years (range)	3.1 (0–14.9)	3.35 (0–14.9)	2.7 (0–10)
Age, no. (%)			
< 18 months	18 (27.7)	11 (19.0)	16 (33.3)
≥ 18 months	47 (72.3)	47 (81.0)	32 (66.7)
Stage, no. (%)			
Localized	30 (46.2)	27 (46.6)	24 (50.0)
Metastatic	35 (53.8)	31 (53.4)	24 (50.0)
Risk group, no. (%)			
High-risk	26 (40.0)	26 (44.8)	16 (33.3)
Non-high risk	39 (60.0)	32 (55.2)	32 (66.7)
Pathology, no. (%)			
Undifferentiated	1 (1.5)	1 (1.7)	1 (2.1)
Poorly differentiated	25 (38.5)	21 (36.2)	19 (39.6)
Differentiating	13 (20.0)	11 (19.0)	9 (18.8)
Ganglioneuroblastoma (GNB)	26 (40.0)	25 (43.1)	19 (39.6)

### Mutation profiles of *MYCN* non-amplified NB

WES data of 58 patients were analysed. The median number of variants per sample was 34.5, with a range of 11–537 (Additional file 1: Figure S1A). Frequently mutated genes were summarized in Additional file 1: Figure S1B. The most frequently mutated gene, *MUC4*, was found to be mutated in 26% of samples, followed by *RBMXL3* (19%), *ALB* (17%), *MUC16* (14%), and *SEPD8* (14%) (Fig. 1b). In comparison with 9 *MYCN*-amplified tumours, there was no statistically significant difference between the mutation frequencies of single genes (Fig. 1c). However, mutations in mucin family genes such as *MUC4*, *MUC16,* and *MUC17* were more frequent in *MYCN* non-amplified subjects.

An association between risk groups and genomic variants was not observed (Additional file 2: Figure S2A). To identify the associations between altered pathways and risk groups, 17,810 annotated gene sets were analysed, and mutation status was determined in each pathway using MSigDB v6.2. Among 7044 pathways acquired from the BIOCARTA, KEGG, REACTOME, and Gene Ontology (GO) databases, 48 pathways had a *P* value < 0.05 in Fisher’s exact test that became insignificant after multiple FDR corrections (Additional file 2: Figure S2B). Alterations in metabolic pathways were enriched in the high-risk group.

### Gene fusions

Gene fusions predicted by three or more algorithms were considered to be true positives. Among 48 WTS samples, 21 gene fusions were detected in 15 samples (Table 2). *CCDC32*-*CBX3* fusion recurred in five samples, while *SAMD5*-*SASH1* fusion recurred in three samples (Additional file 3: Figure S3A). The existence of recurrent fusions was verified by Sanger sequencing. These two recurrent fusions were detected only in ganglioneuroblastoma (GNB) histology. Most of the recurrent fusions were detected in non-high-risk patients, aside from one patient who had four other fusions, including *CCDC32*-*CBX3* and *SAMD5*-*SASH1* fusions. In patients with *SAMD5*-*SASH1* fusion, *SAMD5* and *SASH1* were upregulated; however, this correlation was not observed in *CCDC32*-*CBX3* fusion (Additional file 3: Figure S3B).

**Table 2:** List of patients with gene fusions

Risk group	ID	Sex	Age	Stage	Pathology	Chromosomal abnormality			Event	Fusion
High risk	N_SMC_001	M	3.8	Metastatic	GNB		11q		Recur/Dead	LUC7L3:KLC2
	N_SMC_006	M	3.1	Metastatic	PD		11q		Recur	CNTNAP4:RAB11FIP4
	N_SMC_011	M	3.3	Metastatic	GNB				CCDC32:CBX3* SAMD5:SASH1^$^ HLA-C:HLA-A L1RAPL1:REPS2	
	N_SMC_017	M	2.3	Metastatic	D		11q	17q		PPFIA1:GSDMA
	N_SMC_046	M	5.3	Metastatic	PD	1p				RALGPS2:DNAJC8
	N_SMC_049	M	6.1	Metastatic	PD		11q			RPN1:CCDC58
	N_SMC_070	F	4.6	Metastatic	UD			17q		FBXL7:CDKN3
	N_SMC_076	M	3.8	Metastatic	PD	1p	11q	17q		HP1BP3:NUP85
										MED8:ELOVL1
Non-high risk	N_SMC_003	M	10	Localized	GNB	1p				CCDC32:CBX3*
	N_SMC_026	F	4.1	Localized	GNB					CCDC32:CBX3*
	N_SMC_034	F	2.6	Localized	D			17q		CCDC32:CBX3* KCNH7:MAP3K19 PPP6R2:ANKIB1
	N_SMC_060	M	0.1	Localized	D		11q			MX1:FAM3B
	N_SMC_085	F	2.1	Localized	GNB			17q		CCDC32:CBX3*
	N_SMC_089	M	3.5	Localized	GNB					SAMD5:SASH1^$^
	N_SMC_093	F	6.4	Localized	GNB					SAMD5:SASH1^$^

*UD* undifferentiated, *PD* poorly differentiated, *D* differentiating, *GNB* ganglioneuroblastoma

There are two recurrent fusions *CCDC32:CBX3 ^$^SAMD5:SASH1

### Transcriptome analysis to identify risk-group specific biomarkers

**Fig. 2:**
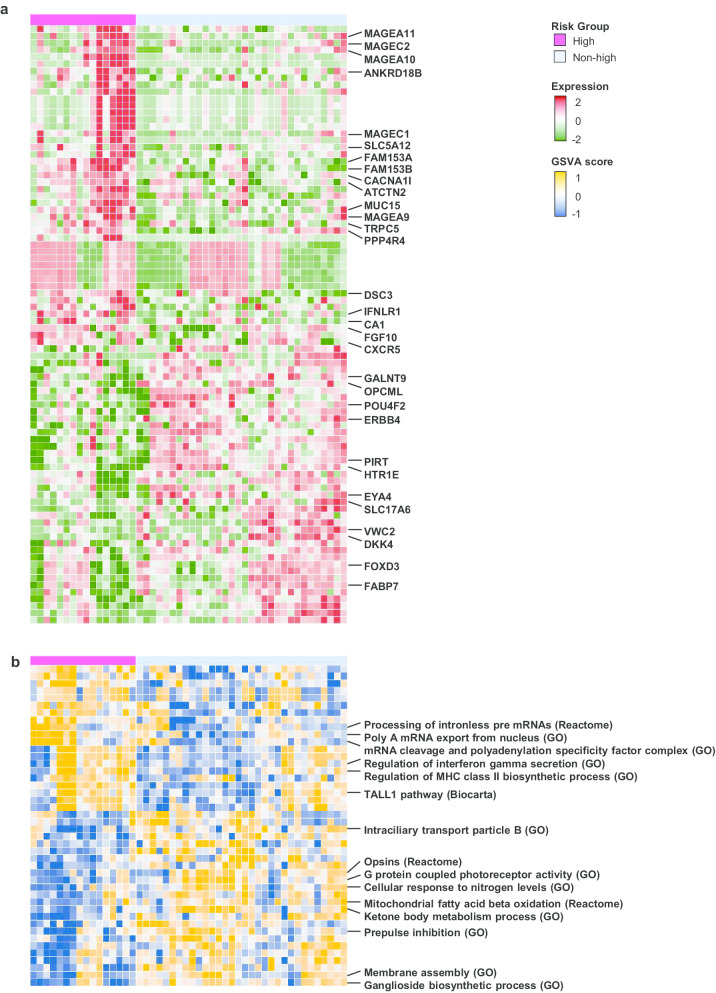
**a** Profiles of differentially expressed genes (n = 86) between high- and non-high-risk groups. **b** GSVA score of gene sets significantly associated with risk group (n = 44)

WTS data of 48 patients showed a correlation between gene expression patterns and the risk-group identity (Additional file 4: Figure S4A). Forty-six genes were significantly over-expressed in the high-risk group and 40 genes were over-expressed in non-high-risk group (*P* value < 0.05 and absolute fold change > 2) (Fig. 2a and Additional file 4: Figure S4B). *FAM153A* (*SAMD15*) and *FAM15B* (*TMED8*) were the most significantly over-expressed genes in the high-risk group. Figure 2 also suggested that there were subgroups in the non-high-risk group, so unsupervised clustering of non-high-risk group was performed. It revealed two distinct subgroups showing clear differences in age, pathologic differentiation and stage (Additional file 4: Figure S4C).

The GSVA score was computed for 17,810 gene sets, and an association test was performed for differentially expressed gene sets between high- and non-high-risk groups (Additional file 5: Figure S5A). In total, 44 gene sets were significantly different between the risk groups (*P* value < 0.05 and absolute mean difference > 0.3) (Fig. 2b and Additional file 5: Figure S5B). The gene sets did not differ in most of the acquired canonical pathways; only 15 of these pathways showed statistically significant differences (Additional file 5: Figure S5C). In the high-risk group, the pathways of ketone body metabolism and mitochondrial fatty acid beta oxidation were inactivated, while the pathways of TALL-1, regulation of MHC class II biosynthesis, and regulation of interferon gamma secretion were activated (Additional file 5: Figure S5D). The ganglioside biosynthesis pathway showed correlations with risk group identity and GNB histology (*P* value = 0.0002) (Additional file 5: Figure S5E).

### Analyses of mutational signature and tumour mutation burden (TMB)

**Fig. 3:**
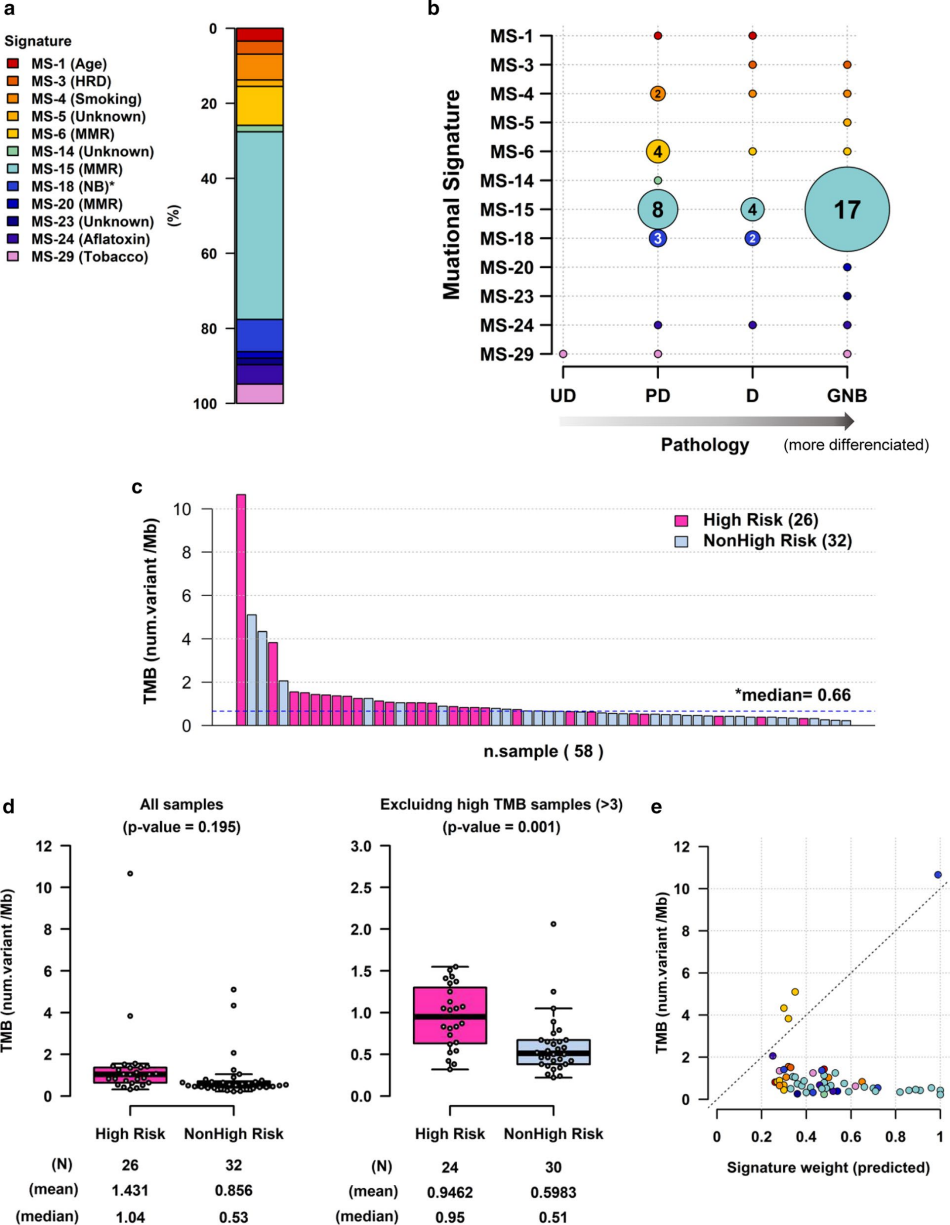
**a** Distribution of predicted mutational signature (MS), which was predominantly presented in each sample. The proportion of samples showing MS-15 (46.6%), MS-6 (13.8%), and MS-18 (8.6%) is presented. **b** Association between signatures and clinical pathology. The size of each circle represents the number of samples. MS-15 and ganglioneuroblastoma (GNB) histology had a significant association (*P* value = 0.0012). **c** Distribution of Tumour Mutation Burden (TMB; n = 58). **d** Comparison of TMB between high-risk (n = 26) and non-high-risk (n = 32) groups with/without high TMB patients (n = 4). **e** Correlation between mutational signature weights and TMB. One hyper-mutated sample (TMB > 10) showed MS-18, and three samples with moderate high TMB showed MS-6

A mutational signature analysis was performed with WES data (Additional file 6: Figure S6A). Each sample was assigned to the most predominant signature among the 30 signatures (MS-1 to MS-30). Sixty percent of samples were assigned to either MS-15 or MS-6, which were denoted as MMR signatures (Fig. 3a). Association was not found between MMR signatures and mutation incidence in MMR-related genes such as *MLH1*, *MSH2/6,* or *PMS2*. Only five samples (5.58%) were assigned to MS-18, a known NB signature. The association between MS-15 and GNB histology was identified (*P* value = 0.0012) (Fig. 3b).

The median TMB was 0.66 Mb (Fig. 3c) in all patients; the specific median values were 1.04 Mb in the high-risk group and 0.53 Mb in the non-high-risk group (*P* value = 0.195). Excluding four outlier samples with TMB > 3 Mb, high-risk patients had significantly higher TMB compared with the non-high-risk patients (0.95 Mb vs 0.60 Mb, *P* value = 0.001) (Fig. 3d). Among the four patients whose tumours had TMB > 3 Mb, two patients belonged to the high-risk group and the other two belonged to the non-high-risk group. One sample from the high-risk group, having two missense mutations (Q1285K and T290K) in *POLE* and one missense mutation (G357W) in *MLH1*, showed a high TMB value of 10.66 Mb. Another high-risk patient with a high TMB value of 3.83 Mb had mutations in *ATR*, *ATRX*, *POLQ*, *RAD54L*, and *SPIDR,* all of which play roles in DNA repair or homologous recombination. Further, two samples from the non-high-risk group showed relatively high TMB, at 5.1 Mb and 4.33 Mb. Both patients were diagnosed at a very young age (under 2 months). Although the tumours of both patients showed high microsatellite instability (MSI), their underlying mutation profiles were different—*POLE* splicing variant was detected in one patient, while the other patient had deleterious mutations in *RAD51AP1*, *RAD51B,* and *RMI2,* which are involved in homologous recombination deficiency.

In terms of the association between TMB and mutational signatures, the sample with extremely high TMB (> 10 Mb) showed MS-18, while the three samples with relatively high TMB (> 3 Mb) showed MS-6, one of the four MMR signatures. (Fig. 3e and Additional file 6: Figure S6B).

## Discussion

The genomic characteristics of *MYCN* non-amplified NB were identified in this study using WES and WTS. *MYCN*-amplified NB was excluded, because *MYCN* amplification is a well-known prognostic factor in NB, and gene expression in *MYCN*-amplified NB is quite different from that in *MYCN*-non-amplified NB. A total of 26 high-risk patients were without *MYCN* amplification, and these patients accounted for 40% of all *MYCN*-non-amplified patients in our dataset.

Common variants of high-risk NB, such as *ALK*, *ATRX*, *PTPN11*, *NRAS,* and *MYCN* mutations [5], were not noticeable. Instead, after eliminating the effect of *MYCN* amplification and considering low-VAF mutations, a number of novel recurrently mutated genes were found. Although the recurrent mutations did not show strong patterns of association with the different risk groups, their roles in *MYCN-*non-amplified NB warrant further exploration. The mutation profiles of *MYCN*-amplified and non-amplified patients were compared, and mutations in mucin family genes were found to be more frequent in the *MYCN*-non-amplified subjects. Although mutations in the mucin gene family have been reported in NB [30], their biological relevance to NB remains unclear.

Two recurrent fusions, *CCDC32*-*CBX3* and *SAMD5*-*SASH1,* were newly detected in this study. No recurrent fusion has been reported in NB, with the exception of fusions including the *NBAS* gene in *MYCN*-amplified tumours [5]. All but one of the patients in this study who presented recurrent fusions fell under the non-high-risk category; thus, it is likely that these fusions have not been reported because most of the previous studies included only high-risk patients. These fusions have been detected in other cancers [31, 32] and further research is needed to investigate the potential roles of these fusions in the tumorigenesis of NB.

In the analysis of risk-specific biomarkers, several genes and gene sets were differentially expressed between the risk groups. Specifically, some immune-related pathways, such as regulation of MHC class II biosynthesis and regulation of interferon gamma secretion, tended to be activated in the high-risk group. In this study, high-risk patients had higher TMB values compared with the non-high-risk patients (when four outlier samples with TMB > 3 Mb were excluded), which could be a factor that causes the activation of immune-related pathways in the high-risk group. The underlying mechanism of this finding remains to be elucidated.

Notably, this study presents several findings in support of the possible application of immunotherapy in NB. In the TMB analysis, a subset of patients was found to have much higher TMB values than the other patients. One sample had TMB > 10 Mb, and three more had a moderate threshold of > 3 Mb. All four of these tumours had mutations in DNA mismatch repair deficiency-related genes or genes involved in homologous recombination deficiency. MSI was high in the two non-high-risk patients. Additionally, in the mutation signature analysis, 65% of tumours showed MMR signatures when each sample was designated to the most predominant signature out of all 30 signatures. PD-1/L1 expression, TMB, and MSI have been considered as predictive biomarkers for immunotherapy in many studies [7, 8, 33–35], and the findings of this study suggest the possibility of immunotherapy introduction in a subset of patients with NB.

Despite comprehensive analysis, this study has several limitations. In mutational signature analysis, MS-18, a known NB signature, was present in only a few samples. Mutational signatures were calculated based on a pattern of 96 base substitution combinations, so an insufficient number of mutations may have affected the analysis. The median number of mutations, at 34.5, was relatively small. Therefore, the mutational signatures of patients with lower numbers of variants may fail to represent all of the characteristics. Since the number of variants and TMB in childhood cancers are smaller compared to those in adult cancers [27, 36–39], the results of mutational signature analysis need to be interpreted with caution. Furthermore, MSigDB contains pathways with large numbers of genes, and the pathways investigated here had gene sets with up to hundreds of genes. Therefore, it is necessary to verify the effects of individual mutations.

## Conclusions

In conclusion, this study provides novel insights into the genomic background of the *MYCN*-non-amplified NB population. Activation of immune-related pathways in the high-risk group and the results of TMB and mutational signature analyses collectively suggested the need for further investigation to discover potential immunotherapeutic strategies for NB.

## Supplementary information

**Additional file 1.** Frequency of variants and mutated genes.

**Additional file 2.** Frequency of mutated genes in risk groups and list of risk group associated pathway.

**Additional file 3.** Sequence of recurrent fusions and expression profile of genes having fusions.

**Additional file 4.** Result of DEG analysis.

**Additional file 5.** Result of GSVA analysis.

**Additional file 6.** Result of Mutational signature analysis.

## Data Availability

The human reference genome sequence was GRCh37-Genome Reference Consortium Human Reference 37(hg19), as downloaded from University of California, Santa Cruz (UCSC) Genome Browser [https://hgdownload.cse.ucsc.edu/goldenpath/hg19/bigZips/]. The neuroblastoma dataset supporting the conclusions of this article is available in the NCBI Sequence Read Archive repository under accession number PRJNA592880, [https://www.ncbi.nlm.nih.gov/sra/PRJNA592880].

## Abbreviations

MMR: Mismatch repair
MS: Mutational signature
MSI: Microsatellite instability
NB: Neuroblastoma
TMB: Tumour mutation burden
WES: Whole exome sequencing
WTS: Whole transcriptome sequencing
