# Supplementary material for: Genomic profile of MYCN non-amplified neuroblastoma and potential for immunotherapeutic strategies in neuroblastoma
Source: BMC Med Genomics. 2020 Nov 10;13:171. doi: 10.1186/s12920-020-00819-5 (PMC7653769; doi:10.1186/s12920-020-00819-5)
Supplement: Supplementary file 1 — Additional file 1. Frequency of variants and mutated genes. [file 12920_2020_819_MOESM1_ESM.pdf]

(A)

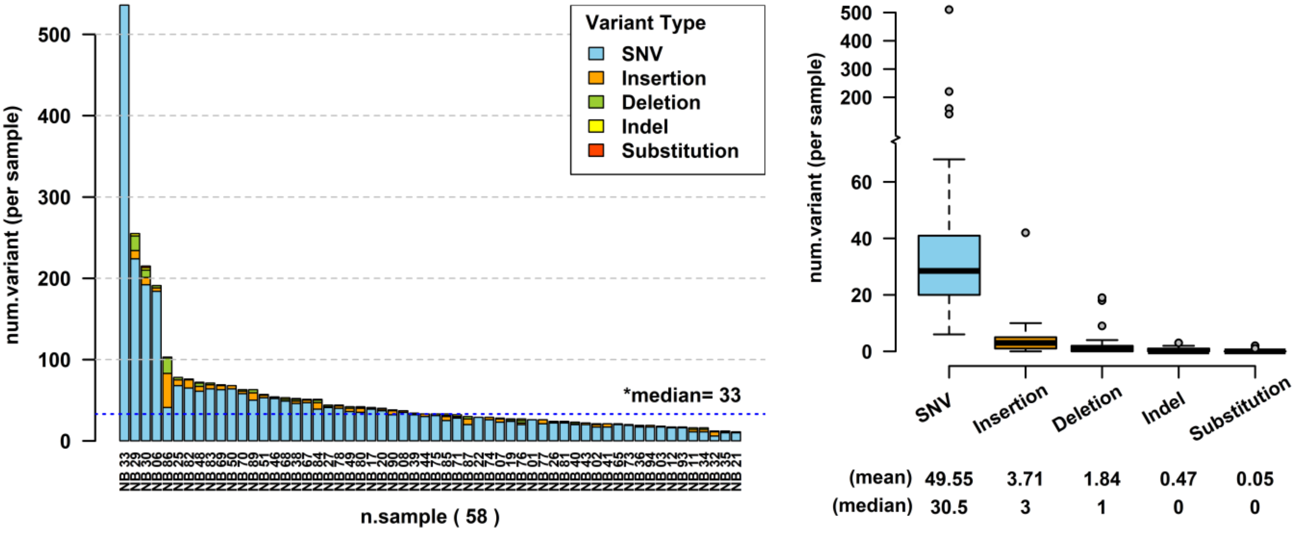

(B)

frequency	n.gene	gene
15	1	MUC4
11	1	RBMXL3
10	1	ALB
8	2	MUC16; SETD8
6	6	AP1M1; MUC17; RP1L1; SLC25A5; TTN; ZNF717
5	8	CSMD1; DNHD1; PABPC1; PABPC3; PLIN4; RPL14; TTR; ZNF208
4	15	APOA1; CTBP2; FAT4; FLG; FSIP2; GPRIN2; HP; KCNMB3; MUC5B; NOP16; OBSCN; PCLO; SKA3; USP6; ZBTB33
3	36	ABCA10; ABCA13; AHNAK; ALDOB; ALK; AMBP; APOH; C3; C5; CARM1; CELA1; EML5; FGG; GGT5; GREB1L; HERC2; HLA-DRB1; HYDIN; KMT2D; LAMA5; LRCH4; MED13; MUC2; MUC3A; OR8U1; PCDH11X; PDHA2; PEAR1; POLE; PRUNE2; RPS3A; SBF2; SLC22A2; STAB2; TANC1; TENM1
2	292	(skip)
1	2239	(skip)
total	2601	(skip)

Figure S1. (A) Distribution of SNV/Indel variants of WES data. (Left) Bar-plot shows the number of variants per sample. The blue line represents the median number of variants; (Right) Box-plot shows the number of variants per sample across variant types. (B) Recurrently mutated gene list from WES data.
