# Supplementary material for: Genomic profile of MYCN non-amplified neuroblastoma and potential for immunotherapeutic strategies in neuroblastoma
Source: BMC Med Genomics. 2020 Nov 10;13:171. doi: 10.1186/s12920-020-00819-5 (PMC7653769; doi:10.1186/s12920-020-00819-5)
Supplement: Supplementary file 2 — Additional file 2. Frequency of mutated genes in risk groups and list of risk group associated pathway. [file 12920_2020_819_MOESM2_ESM.pdf]

(A)

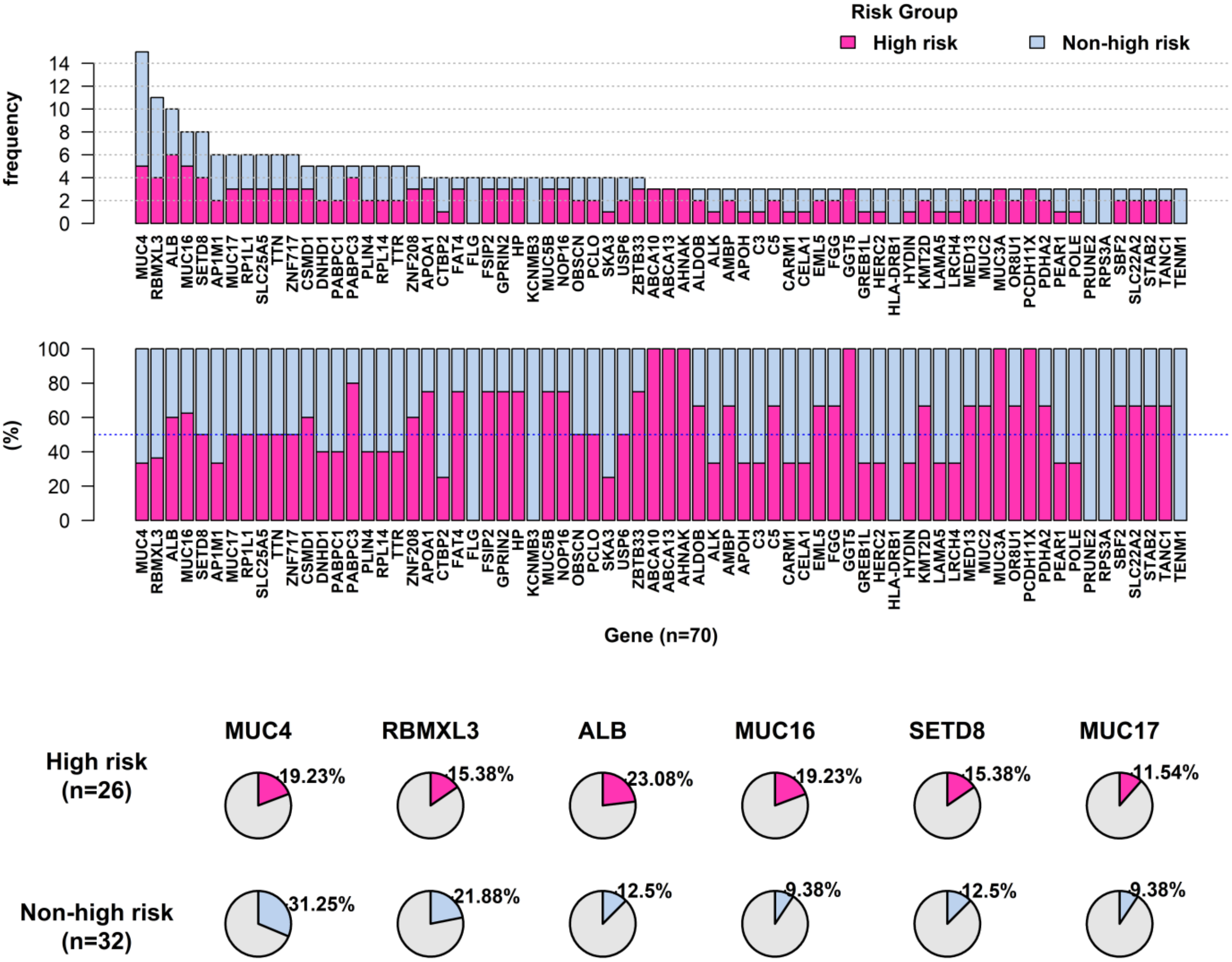

Figure S2. (A) The frequency of mutated genes in risk groups. The proportion of high/non-high risk groups is presented for each mutated gene to identify the association between mutation and risk group. Significant difference in the frequency of mutations between high and non-high risk group was not observed.

(B)

Geneset Name	WT High risk	WT Non-high risk	MUT High risk	MUT Non-high risk	P-value
GO_ATPASE_ACTIVITY_COUPLED	5	22	21	10	0.00021
GO_PROTEIN_MODIFICATION_BY_SMALL_PROTEIN_CONJUGATION_OR_REMOVAL	0	12	26	20	0.00026
GO_TRANSCRIPTIONAL_ACTIVATOR_ACTIVITY_RNA_POLYMERASE_II_CORE_PROMOTER_PROXIMAL_REGION_SEQUENCE_SPECIFIC_BINDING	12	29	14	3	0.00034
GO_MITOCHONDRIAL_MATRIX	8	25	18	7	0.00046
GO_REGULATION_OF_PROTEIN_MODIFICATION_BY_SMALL_PROTEIN_CONJUGATION_OR_REMOVAL	11	28	15	4	0.00053
GO_POSITIVE_REGULATION_OF_PROTEIN_MODIFICATION_BY_SMALL_PROTEIN_CONJUGATION_OR_REMOVAL	14	30	12	2	0.00055
GO_REGULATION_OF_MUSCLE_ADAPTATION	18	32	8	0	0.00082
GO_REGULATION_OF_SYSTEMIC_ARTERIAL_BLOOD_PRESSURE_BY_HORMONE	18	32	8	0	0.00082
GO_ENDOCRINE_PROCESS	18	32	8	0	0.00082
GO_REGULATION_OF_SYSTEMIC_ARTERIAL_BLOOD_PRESSURE_MEDIATED_BY_A_CHEMICAL_SIGNAL	18	32	8	0	0.00082
GO_ATPASE_ACTIVITY_COUPLED_TO_MOVEMENT_OF_SUBSTANCES	13	29	13	3	0.00090
GO_RESPONSE_TO_WOUNDING	3	17	23	15	0.00100
GO_TRANSCRIPTION_FROM_RNA_POLYMERASE_II_PROMOTER	3	17	23	15	0.00100
GO_DEFENSE_RESPONSE_TO_OTHER_ORGANISM	3	17	23	15	0.00100
GO_REGULATION_OF_IMMUNE_EFFECTOR_PROCESS	3	17	23	15	0.00100
GO_TRANSCRIPTION_FACTOR_ACTIVITY_RNA_POLYMERASE_II_CORE_PROMOTER_PROXIMAL_REGION_SEQUENCE_SPECIFIC_BINDING	10	26	16	6	0.00115
GO_PROTEIN_UBIQUITINATION	2	15	24	17	0.00128
GO_CELL_ACTIVATION	5	20	21	12	0.00131
GO_TRANSCRIPTION_FACTOR_BINDING	5	20	21	12	0.00131
**GO_UNSATURATED_FATTY_ACID_METABOLIC_PROCESS**	16	31	10	1	0.00135
**GO_FATTY_ACID_DERIVATIVE_METABOLIC_PROCESS**	16	31	10	1	0.00135
GO_REGULATION_OF_DENDRITE_MORPHOGENESIS	15	30	11	2	0.00143
GO_TRANSCRIPTIONAL_ACTIVATOR_ACTIVITY_RNA_POLYMERASE_II_TRANSCRIPTION_REGULATORY_REGION_SEQUENCE_SPECIFIC_BINDING	11	27	15	5	0.00184
GO_LIPID_BINDING	3	16	23	16	0.00218
GO_EXCRETION	19	32	7	0	0.00219
GO_POSITIVE_REGULATION_OF_HEART_GROWTH	19	32	7	0	0.00219
GO_POSITIVE_REGULATION_OF_TRANSCRIPTION_FROM_RNA_POLYMERASE_II_PROMOTER_IN_RESPONSE_TO_STRESS	19	32	7	0	0.00219
GO_POSITIVE_REGULATION_OF_TRANSCRIPTION_FROM_RNA_POLYMERASE_II_PROMOTER_INVOLVED_IN_CELLULAR_RESPONSE_TO_CHEMICAL_STIMULUS	19	32	7	0	0.00219
GO_POSITIVE_REGULATION_OF_ORGAN_GROWTH	19	32	7	0	0.00219
GO_REGULATION_OF_BLOOD_PRESSURE	14	29	12	3	0.00223
GO_B_CELL_ACTIVATION	14	29	12	3	0.00223
**GO_NEGATIVE_REGULATION_OF_DNA_METABOLIC_PROCESS**	14	29	12	3	0.00223
GO_REGULATION_OF_NEURAL_PRECURSOR_CELL_PROLIFERATION	14	29	12	3	0.00223
GO_PURINE_NTP_DEPENDENT_HELICASE_ACTIVITY	14	29	12	3	0.00223
GO_CELLULAR_CATABOLIC_PROCESS	0	9	26	23	0.00293
**GO_MONOCARBOXYLIC_ACID_METABOLIC_PROCESS**	5	19	21	13	0.00301
REACTOME_INTEGRIN_CELL_SURFACE_INTERACTIONS	10	25	16	7	0.00305
GO_SYNAPSE_ORGANIZATION	10	25	16	7	0.00305
GO_ALCOHOL_BINDING	13	28	13	4	0.00317
**GO_FATTY_ACID_METABOLIC_PROCESS**	9	24	17	8	0.00319
GO_REGULATION_OF_DNA_TEMPLATED_TRANSCRIPTION_IN_RESPONSE_TO_STRESS	17	31	9	1	0.00325
GO_CELL_CELL_ADHESION_VIA_PLASMA_MEMBRANE_ADHESION_MOLECULES	8	23	18	9	0.00327
GO_REGULATION_OF_CELLULAR_COMPONENT_BIOGENESIS	1	12	25	20	0.00329
GO_CORE_PROMOTER_PROXIMAL_REGION_DNA_BINDING	7	22	19	10	0.00330
KEGG_BASAL_CELL_CARCINOMA	16	30	10	2	0.00350
GO_CULLIN_RING_UBIQUITIN_LIGASE_COMPLEX	16	30	10	2	0.00350
GO_SENSORY_ORGAN_MORPHOGENESIS	7	21	19	11	0.00423
GO_REGULATION_OF_DENDRITE_DEVELOPMENT	12	27	14	5	0.00423

Figure S2. (B) List of 48 pathways whose alteration is associated with risk groups. By categorizing mutation status in each pathway, the association between altered pathways and risk groups is examined. The significance of association was determined by Fisher’s exact test.
