# Supplementary material for: Genomic profile of MYCN non-amplified neuroblastoma and potential for immunotherapeutic strategies in neuroblastoma
Source: BMC Med Genomics. 2020 Nov 10;13:171. doi: 10.1186/s12920-020-00819-5 (PMC7653769; doi:10.1186/s12920-020-00819-5)
Supplement: Supplementary file 3 — Additional file 3. Sequence of recurrent fusions and expression profile of genes having fusions. [file 12920_2020_819_MOESM3_ESM.pdf]

(A)

Recurrent Fusion	Target fusion junction sequence
**CCDC32-CBX3**	CTCAGATTCCAAATGAAAATGTTTGAGAGCGCTGACTCTACAGCCACAAGATCTGGCCAGGATCTCTGGG<br>CTGAAATTTGTTCCTGTCTGCCAAATCCTGAACAAGAAGATGGTGCCAACAATGCATTCTCAGACTCCTTT<br>GTGGATTCTTGCCCTGAAGGTGAAGGCCAGAGGGAGGTGGCTGACTTTGCTGTCCAGCCAGCTGTAAAG<br>CCTTGGGCTCCCTTGC**AGGATTCAGAAGTGTATTTAGCATCTCTAGCCATTTTATTTTAAAAATATTTCCTG**<br>**ACTTCGGATGTGGCTTGAGCTGTAG GCGCGGAGGGCCGGAGACGCTGCAGACCCG**CGACCCGGAGCA<br>GCTCGGAGGCGGTGAATAATAGCTCTTCAAGTCTGCAATAAAAAATGGCCTCCAACAAAACTACATTGCAA<br>AAAATGGGAAAAAAACAGAATGGAAAGAGTAAAAAAGTTGAAGAGGCAGAGCCTGAAGAATTTGTCGTGG<br>AAAAAGTACTAGATCGACGTGTAGTGAATGGGAAAGTGGAATATTTCCTGAAGTGGAAGGGATTTACAGAT<br>GCTGACAATACTTGGGAACCTGAAGAAAATTTAGATTGTCCAGAATTGATTGAAGCGTTTCTTAACTCTCAG<br>AAAGCTGGCAAAGAAAAAGATGGTACAAAAAGAAAATC
**SAMD5-SASH1**	CGGGAGCAGGACGCCAACGCCGCCGGCCTCTACTTCACGCTTGAGCCGCAGCCGGCGCCCCCCGGGC<br>CGCCCGCCGACGCCGTCCCCACCGGCCGCCGGGGGGAGCCGTGCGGCGGCCCGGCCCAGGGCACCC<br>GCGGGGACTCTCGCGGCCACACGACCGCCCCCCGCAGCAGGGAGCTGGTGAGCTACCCCAAACTGAA<br>GCTGAAGATCATGATCAGGGATAAGCTCGTCCGTGACGGCATC**CACCTGAGCAAGCCCCCGTACTCCCG**<br>**CAAGGACGGTTCA**CTGGGAAACATCGATGACCTGGCGCAGCAGTATGCAGATTATTACAACACCTGTTTC<br>TCCGACGTGTGCGAGAGGATGGAGGAGCTGCGGAAACGGCGGGTTTCCCAGGACCTGGAAGTGGAGAA<br>ACCCGATGCTAGCCCCACGTCACTTCAGCTGCGGTCCCAGATCGAAGAGTCGCTTGGCTTCTGTAGCGC<br>CGTGTCAACCCCAGAAGTGGAAAGAAAGAACCCTCTTCATAAATCAAACTCAGAAGACAGCTCTGTAGGA<br>AAAGGAGACT

(B)

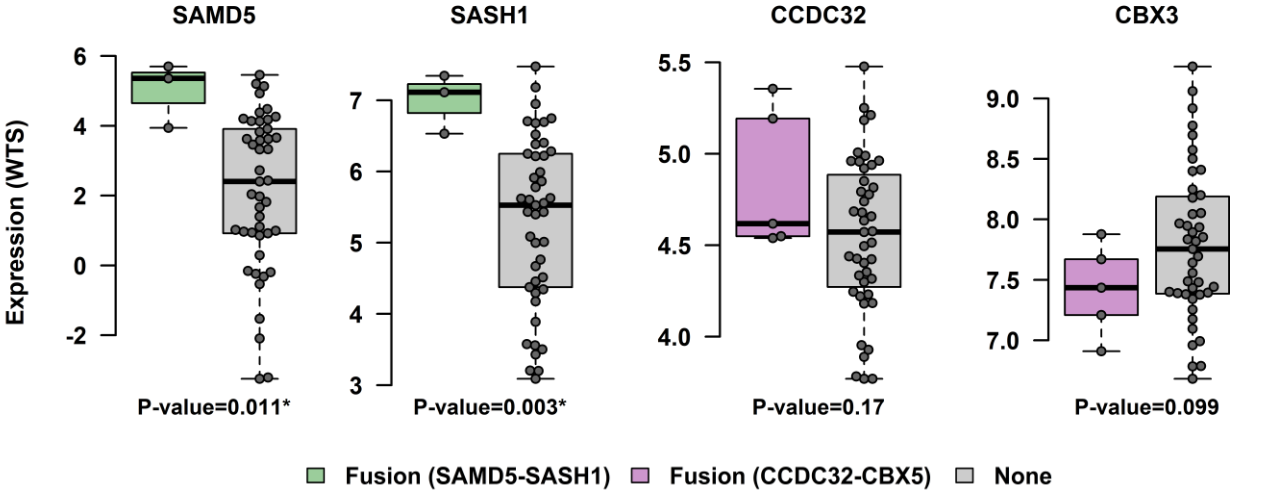

Figure S3. (A) The junction sequence of two recurrent fusions. (B) Expression profiles of genes having SMAD5-SASH1 or CCDC32-CBX3 fusion. Expression level of SASH1 and SAMD5 genes is higher in patients with SAMD5-SASH1 fusion.
