# Supplementary material for: Genomic profile of MYCN non-amplified neuroblastoma and potential for immunotherapeutic strategies in neuroblastoma
Source: BMC Med Genomics. 2020 Nov 10;13:171. doi: 10.1186/s12920-020-00819-5 (PMC7653769; doi:10.1186/s12920-020-00819-5)
Supplement: Supplementary file 4 — Additional file 4. Result of DEG analysis. [file 12920_2020_819_MOESM4_ESM.pdf]

(A)

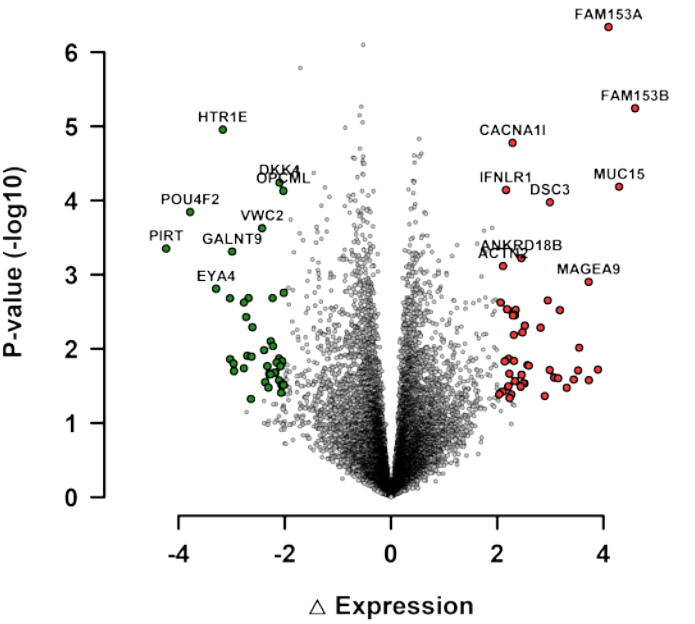

A total of 86 genes were significant. (red/green dot)
* Threshold for significance: |△ Expression| > 2 & P-value <0.05

(B)

Class	Gene	High risk (avg expr.)	Non-high risk (avg expr.)	diff.expr	T-stat.	P-value	P-value (FDR)
**Overexpressed in High risk**	**FAM153A**	1.780	-2.318	4.098	6.413	0.000	**0.006**
	**FAM153B**	1.677	-2.924	4.601	5.681	0.000	**0.018**
	**CACNA1I**	1.577	-0.715	2.292	4.889	0.000	**0.023**
	**MUC15**	0.632	-3.666	4.298	4.778	0.000	**0.032**
	**IFNLR1**	1.613	-0.554	2.167	4.743	0.000	**0.032**
	**DSC3**	0.446	-2.548	2.994	4.276	0.000	**0.037**
	**ANKRD18B**	-1.714	-4.172	2.459	3.998	0.001	0.070
	**ACTN2**	4.811	2.699	2.113	3.637	0.001	0.077
	**MAGEA9**	0.847	-2.876	3.723	3.693	0.001	0.093
	**MAGEC1**	-1.120	-4.075	2.954	3.413	0.002	0.115
	**PPP4R4**	1.226	-0.842	2.068	3.221	0.002	0.117
	**SLC5A12**	0.328	-1.861	2.189	3.332	0.003	0.129
	**CRB2**	-0.162	-2.513	2.352	3.357	0.003	0.129
	**POTEM**	-2.020	-5.204	3.184	3.484	0.003	0.129
	**TRPC5**	-0.531	-2.867	2.336	3.146	0.004	0.138
	**LGSN**	-0.421	-2.720	2.299	3.210	0.004	0.138
	**ALX3**	-1.354	-3.878	2.524	3.090	0.005	0.161
	**SLCO1A2**	0.773	-2.048	2.821	3.077	0.005	0.166
	**OR4N4**	-2.959	-5.437	2.478	3.154	0.006	0.176
	**SAA1**	-0.878	-3.193	2.316	2.922	0.007	0.181
	**MAGEA4**	-0.256	-3.801	3.545	2.858	0.010	0.216
	**NXF2B**	0.226	-1.991	2.217	2.588	0.014	0.249
	**CXCR5**	1.524	-0.795	2.320	2.565	0.015	0.255
	**CCDC144NL**	1.252	-0.891	2.143	2.617	0.015	0.255
	**TUBA3C**	-2.624	-5.197	2.573	2.670	0.017	0.266
	**WDR72**	-1.554	-4.152	2.598	2.633	0.017	0.266
	**DDX3Y**	3.472	-0.426	3.898	2.466	0.019	0.281
	**MAGEA11**	-0.376	-3.367	2.991	2.536	0.019	0.281
	**KDM5D**	2.707	-0.816	3.523	2.459	0.020	0.283
	**FGF10**	1.859	-0.371	2.229	2.406	0.022	0.293
	**NAA11**	-3.193	-5.655	2.461	2.538	0.023	0.298
	**MAGEA10**	1.807	-1.270	3.077	2.371	0.025	0.306
	**UTY**	1.430	-1.718	3.148	2.350	0.025	0.308
	**USP9Y**	2.468	-0.977	3.445	2.331	0.026	0.315
	**RPS4Y1**	4.865	1.135	3.730	2.318	0.027	0.316
	**XAGE1A**	-2.755	-5.090	2.335	2.428	0.027	0.320
	**XAGE1D**	-2.199	-4.714	2.515	2.389	0.029	0.327
	**XAGE1E**	-2.597	-5.090	2.493	2.390	0.030	0.328
	**CA1**	-0.849	-3.064	2.215	2.280	0.032	0.335
	**XAGE1C**	-2.595	-5.040	2.446	2.343	0.032	0.337
	**EIF1AY**	2.196	-1.116	3.312	2.217	0.034	0.341
	**IGLL5**	3.693	1.590	2.103	2.204	0.038	0.354
	**NMU**	-1.607	-3.649	2.042	2.182	0.041	0.365
	**XAGE1B**	-2.386	-4.656	2.270	2.218	0.041	0.366
	**ZFY**	1.430	-1.469	2.899	2.105	0.043	0.374
	**MAGEC2**	-1.671	-3.907	2.235	2.118	0.046	0.383

Class	Gene	High risk (avg expr.)	Non-high risk (avg expr.)	diff.expr	T-stat.	P-value	P-value (FDR)
**Underexpressed in High risk**	**HTR1E**	-3.903	-0.737	-3.166	-5.212	0.000	**0.020**
	**DKK4**	-4.904	-2.803	-2.101	-4.434	0.000	**0.030**
	**OPCML**	-0.608	1.417	-2.025	-4.670	0.000	**0.032**
	**POU4F2**	-3.866	-0.083	-3.783	-4.271	0.000	**0.040**
	**VWC2**	-4.768	-2.343	-2.425	-3.988	0.000	**0.050**
	**PIRT**	1.015	5.246	-4.231	-4.063	0.000	0.068
	**GALNT9**	-2.191	0.799	-2.989	-3.988	0.000	0.068
	**EYA4**	-1.373	1.918	-3.292	-3.454	0.002	0.100
	**ERBB4**	-2.470	-0.452	-2.018	-3.418	0.002	0.104
	**SLC17A6**	-4.305	-2.075	-2.229	-3.265	0.002	0.113
	**GALR1**	-3.692	-1.009	-2.683	-3.411	0.002	0.113
	**SPHKAP**	-2.521	0.512	-3.033	-3.418	0.002	0.113
	**SLITRK3**	-3.480	-0.714	-2.766	-3.306	0.002	0.117
	**CRABP1**	-1.797	0.929	-2.726	-3.071	0.004	0.141
	**EYA1**	0.429	3.036	-2.607	-3.042	0.005	0.165
	**NLGN1**	0.082	2.344	-2.262	-2.960	0.008	0.200
	**DIO3**	-1.368	0.854	-2.222	-2.778	0.009	0.212
	**CSAG1**	-3.097	-0.710	-2.387	-2.784	0.010	0.222
	**FABP7**	-1.346	1.360	-2.705	-2.726	0.012	0.242
	**NELL1**	-2.484	0.142	-2.626	-2.715	0.013	0.245
	**DGKB**	1.760	3.869	-2.109	-2.719	0.014	0.249
	**ADCYAP1**	2.578	5.604	-3.026	-2.614	0.014	0.251
	**PRND**	-0.032	2.017	-2.049	-2.628	0.015	0.255
	**INSM1**	-0.058	2.094	-2.152	-2.530	0.015	0.259
	**CACNG7**	-1.230	1.732	-2.962	-2.592	0.016	0.261
	**HOXC10**	-1.062	1.265	-2.326	-2.562	0.017	0.267
	**TMEFF2**	1.963	4.041	-2.078	-2.551	0.017	0.268
	**SLC5A7**	-2.869	-0.101	-2.768	-2.483	0.018	0.276
	**DLK1**	1.573	4.527	-2.954	-2.505	0.020	0.286
	**ARHGDIG**	2.421	4.598	-2.177	-2.515	0.021	0.289
	**MAGEA2B**	-4.671	-2.477	-2.194	-2.424	0.021	0.289
	**SPOCK3**	1.061	3.353	-2.292	-2.461	0.022	0.294
	**MIA**	1.669	3.938	-2.269	-2.409	0.022	0.295
	**CLDN19**	-3.622	-1.514	-2.108	-2.302	0.027	0.318
	**CGA**	-3.052	-0.683	-2.369	-2.285	0.028	0.323
	**SV2C**	1.154	3.213	-2.058	-2.303	0.031	0.331
	**GABRG1**	-1.989	0.035	-2.024	-2.273	0.031	0.331
	**NKAIN4**	-1.127	1.179	-2.306	-2.265	0.033	0.341
	**CDH10**	-0.579	1.488	-2.067	-2.177	0.039	0.360
	**FOXD3**	0.190	2.827	-2.637	-2.089	0.048	0.386

Figure S4. (A) Volcano plot representing the result of DEG (n = 86). Red and green dots indicate significantly over or under expressed genes, respectively, in high risk group. (B) List of DEGs (n = 86). While 46 genes were overexpressed, 40 genes were under-expressed, in high risk group. The significance was determined by two-group *T*-test (P-value < 0.05) and difference of average expression between risk-groups > 2.

(C)

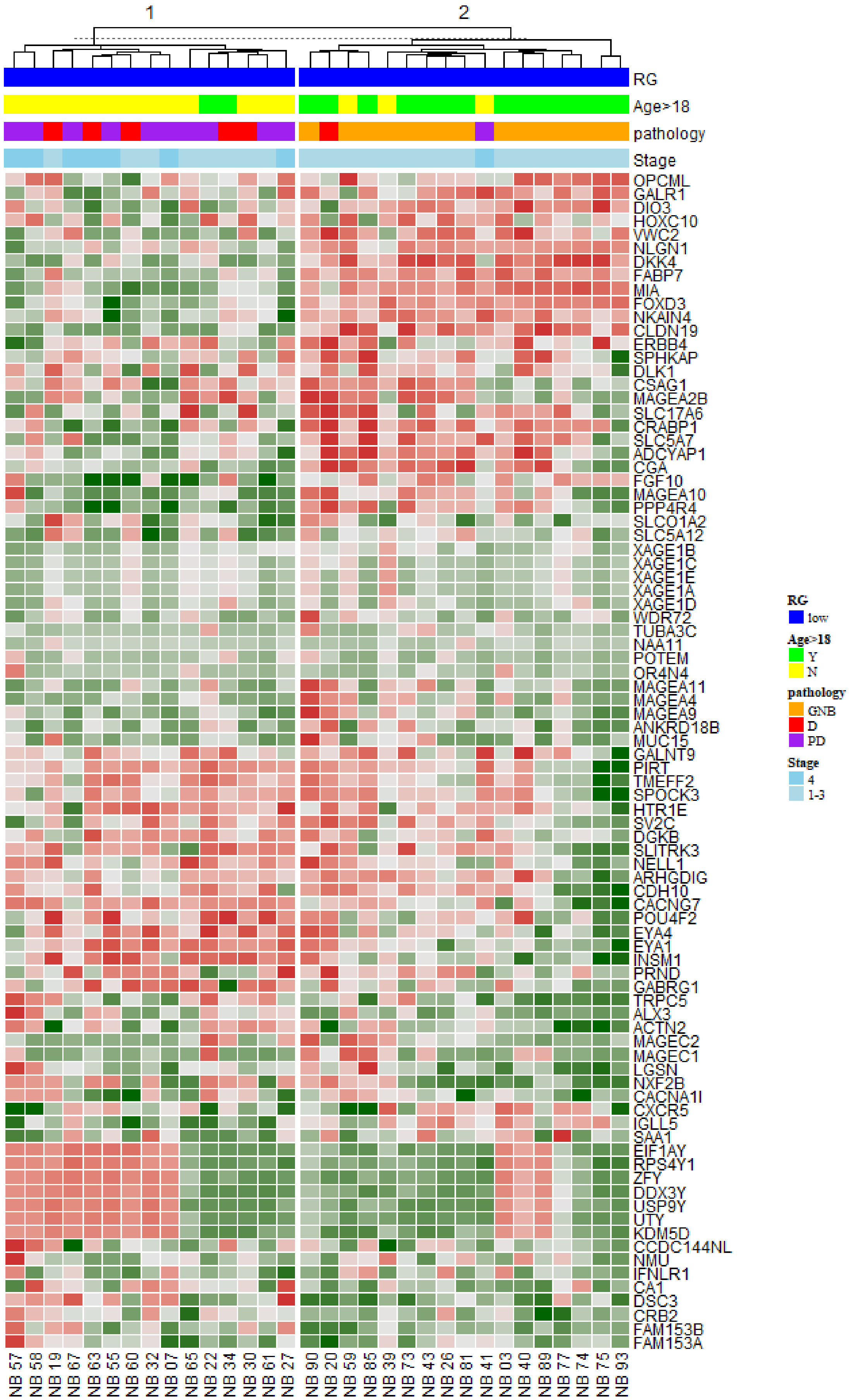

Figure S4. (C) Unsupervised clustering of non-high-risk group. Unsupervised clustering of non-high-risk group revelaed two distinct subgroups showing clear differences in age, pathologic differentiation and stage.
