# Supplementary material for: Genomic profile of MYCN non-amplified neuroblastoma and potential for immunotherapeutic strategies in neuroblastoma
Source: BMC Med Genomics. 2020 Nov 10;13:171. doi: 10.1186/s12920-020-00819-5 (PMC7653769; doi:10.1186/s12920-020-00819-5)
Supplement: Supplementary file 5 — Additional file 5. Result of GSVA analysis. [file 12920_2020_819_MOESM5_ESM.pdf]

(A)

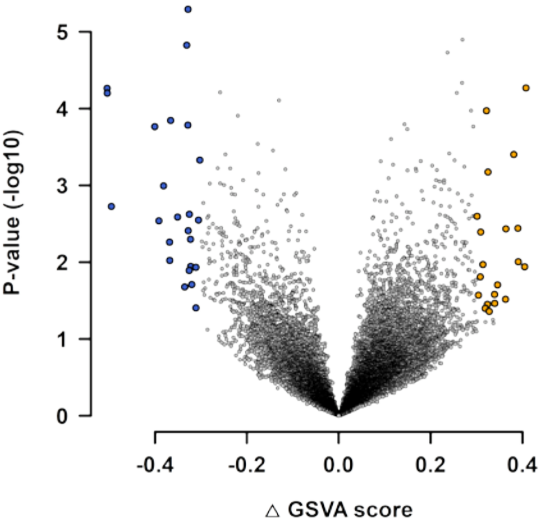

A total of 44 genesets were significant. (orange/blue dot)
* Threshold for significance: |△ GSVA score| > 0.3 & P-value <0.05

(B)

Class	Gene set name	High risk (avg GSVA score)	Non-high risk (avg GSVA score)	diff.score	T-stat.	P-value	FDR
**Activated In high risk group**	CROONQUIST_STROMAL_STIMULATION_DN	0.278	-0.130	0.408	4.604	0.000	0.112
	INAMURA_LUNG_CANCER_SCC_SUBTYPES_UP	0.175	-0.147	0.322	4.346	0.000	0.147
	chr9p	0.263	-0.118	0.381	3.866	0.000	0.271
	BALLIF_DEVELOPMENTAL_DISABILITY_P16_P12_DELETION	0.214	-0.111	0.325	3.814	0.001	0.292
	GO_POLY_A_MRNA_EXPORT_FROM_NUCLEUS	0.132	-0.170	0.301	3.321	0.003	0.452
	INAMURA_LUNG_CANCER_SCC_SUBTYPES_DN	0.251	-0.139	0.390	3.095	0.004	0.499
	chr19p	0.206	-0.158	0.364	3.122	0.004	0.499
	BIOCARTA_TALL1_PATHWAY	0.215	-0.094	0.309	3.095	0.004	0.499
	BRUNEAU_SEPTATION_ATRIAL	0.272	-0.119	0.391	2.722	0.010	0.609
	GO_REGULATION_OF_MHC_CLASS_II_BIOSYNTHETIC_PROCESS	0.220	-0.094	0.314	2.759	0.011	0.627
	chr18p	0.297	-0.108	0.405	2.682	0.011	0.635
	GO_MRNA_CLEAVAGE_AND_POLYADENYLATION_SPECIFICITY_FACTOR_COMPLEX	0.111	-0.198	0.308	2.595	0.016	0.690
	LI_WILMS_TUMOR_ANAPLASTIC_DN	0.251	-0.095	0.346	2.471	0.020	0.703
	GO_REGULATION_OF_INTERFERON_GAMMA_SECRETION	0.239	-0.100	0.340	2.382	0.026	0.734
	REACTOME_PROCESSING_OF_INTRONLESS_PRE_MRNAS	0.093	-0.211	0.304	2.349	0.027	0.735
	RUNNE_GENDER_EFFECT_UP	0.251	-0.112	0.364	2.261	0.031	0.748
	FINETTI_BREAST_CANCER_KINOME_GREEN	0.259	-0.081	0.340	2.239	0.035	0.750
	MODULE_73	0.241	-0.083	0.324	2.221	0.036	0.751
	BASSO_HAIRY_CELL_LEUKEMIA_DN	0.188	-0.131	0.319	2.150	0.040	0.760
	chryq11	0.231	-0.097	0.328	2.090	0.044	0.763
**Inactivated In high risk group**	chr7p21	-0.238	0.090	-0.328	-5.267	0.000	0.083
	chr17p12	-0.222	0.109	-0.331	-4.941	0.000	0.083
	GO_G_PROTEIN_COUPLED_PHOTORECEPTOR_ACTIVITY	-0.303	0.201	-0.504	-4.605	0.000	0.112
	HESSON_TUMOR_SUPPRESSOR_CLUSTER_3P21_3	-0.340	0.164	-0.504	-4.708	0.000	0.112
	TESAR_ALK_TARGETS_EPISC_3D_UP	-0.249	0.117	-0.366	-4.322	0.000	0.161
	chr6q13	-0.179	0.150	-0.328	-4.268	0.000	0.161
	chr11q25	-0.286	0.114	-0.400	-4.311	0.000	0.161
	GO_GANGLIOSIDE_BIOSYNTHETIC_PROCESS	-0.174	0.128	-0.302	-4.083	0.000	0.288
	GO_KETONE_BODY_METABOLIC_PROCESS	-0.259	0.122	-0.381	-3.579	0.001	0.348
	REACTOME_OPSINS	-0.278	0.217	-0.495	-3.383	0.002	0.406
	MIKHAYLOVA_OXIDATIVE_STRESS_RESPONSE_VIA_VHL_UP	-0.218	0.107	-0.325	-3.228	0.002	0.453
	NIKOLSKY_BREAST_CANCER_17P11_AMPLICON	-0.208	0.142	-0.351	-3.302	0.003	0.453
	GO_PREPULSE_INHIBITION	-0.154	0.152	-0.305	-3.300	0.003	0.467
	VANDESLUIS_NORMAL_EMBRYOS_UP	-0.264	0.128	-0.391	-3.193	0.003	0.467
	GO_INTRACILIARY_TRANSPORT_PARTICLE_B	-0.220	0.107	-0.327	-3.154	0.004	0.499
	SCHAEFFER_PROSTATE_DEVELOPMENT_AND_CANCER_BOX5_DN	-0.191	0.131	-0.323	-3.004	0.005	0.534
	chr6q26	-0.189	0.179	-0.368	-2.984	0.006	0.534
	REACTOME_MITOCHONDRIAL_FATTY_ACID_BETA_OXIDATION	-0.232	0.136	-0.368	-2.777	0.010	0.601
	CASTELLANO_HRAS_TARGETS_UP	-0.238	0.085	-0.322	-2.663	0.011	0.634
	GO_MEMBRANE_ASSEMBLY	-0.155	0.155	-0.311	-2.716	0.012	0.640
	MODULE_101	-0.223	0.102	-0.325	-2.629	0.013	0.656
	GO_CELLULAR_RESPONSE_TO_NITROGEN_LEVELS	-0.111	0.209	-0.320	-2.466	0.020	0.702
	CLIMENT_BREAST_CANCER_COPY_NUMBER_DN	-0.156	0.179	-0.335	-2.473	0.021	0.714
	CHEN_HOXA5_TARGETS_6HR_DN	-0.245	0.066	-0.311	-2.155	0.040	0.757

Figure S5. (A) Volcano plot representing the result of GSVA. Orange and blue dots indicate significantly activated and inactivated gene-sets, respectively, in high risk group. (B) Among 17,810 gene sets from MsigDB, a total of 44 gene sets showed significant association with the risk group. The significance was determined by two-group *T*-test (P-value < 0.05) and difference of average GSVA score between risk-groups > 0.3.

(C)

Class	Gene set name	High risk (avg. GSVA score)	Non-high risk (avg. GSVA score)	diff.score	T-stat.	P-value
Activated in high risk	GO_POLY_A_MRNA_EXPORT_FROM_NUCLEUS	0.132	-0.170	0.301	3.321	0.003
	REACTOME_PROCESSING_OF_INTRONLESS_PRE_MRNAS	0.093	-0.211	0.304	2.349	0.027
	GO_MRNA_CLEAVAGE_AND_POLYADENYLATION_SPECIFICITY_FACTOR_COMPLEX	0.111	-0.198	0.308	2.595	0.016
	BIOCARTA_TALL1_PATHWAY	0.215	-0.094	0.309	3.095	0.004
	GO_REGULATION_OF_MHC_CLASS_II_BIOSYNTHETIC_PROCESS	0.220	-0.094	0.314	2.759	0.011
	GO_REGULATION_OF_INTERFERON_GAMMA_SECRETION	0.239	-0.100	0.340	2.382	0.026
Inactivated in high risk	GO_G_PROTEIN_COUPLED_PHOTORECEPTOR_ACTIVITY	-0.303	0.201	-0.504	-4.605	0.000
	REACTOME_OPSINS	-0.278	0.217	-0.495	-3.383	0.002
	GO_KETONE_BODY_METABOLIC_PROCESS	-0.259	0.122	-0.381	-3.579	0.001
	REACTOME_MITOCHONDRIAL_FATTY_ACID_BETA_OXIDATION	-0.232	0.136	-0.368	-2.777	0.010
	GO_INTRACILIARY_TRANSPORT_PARTICLE_B	-0.220	0.107	-0.327	-3.154	0.004
	GO_CELLULAR_RESPONSE_TO_NITROGEN_LEVELS	-0.111	0.209	-0.320	-2.466	0.020
	GO_MEMBRANE_ASSEMBLY	-0.155	0.155	-0.311	-2.716	0.012
	GO_PREPULSE_INHIBITION	-0.154	0.152	-0.305	-3.300	0.003
	GO_GANGLIOSIDE_BIOSYNTHETIC_PROCESS	-0.174	0.128	-0.302	-4.083	0.000

(D)

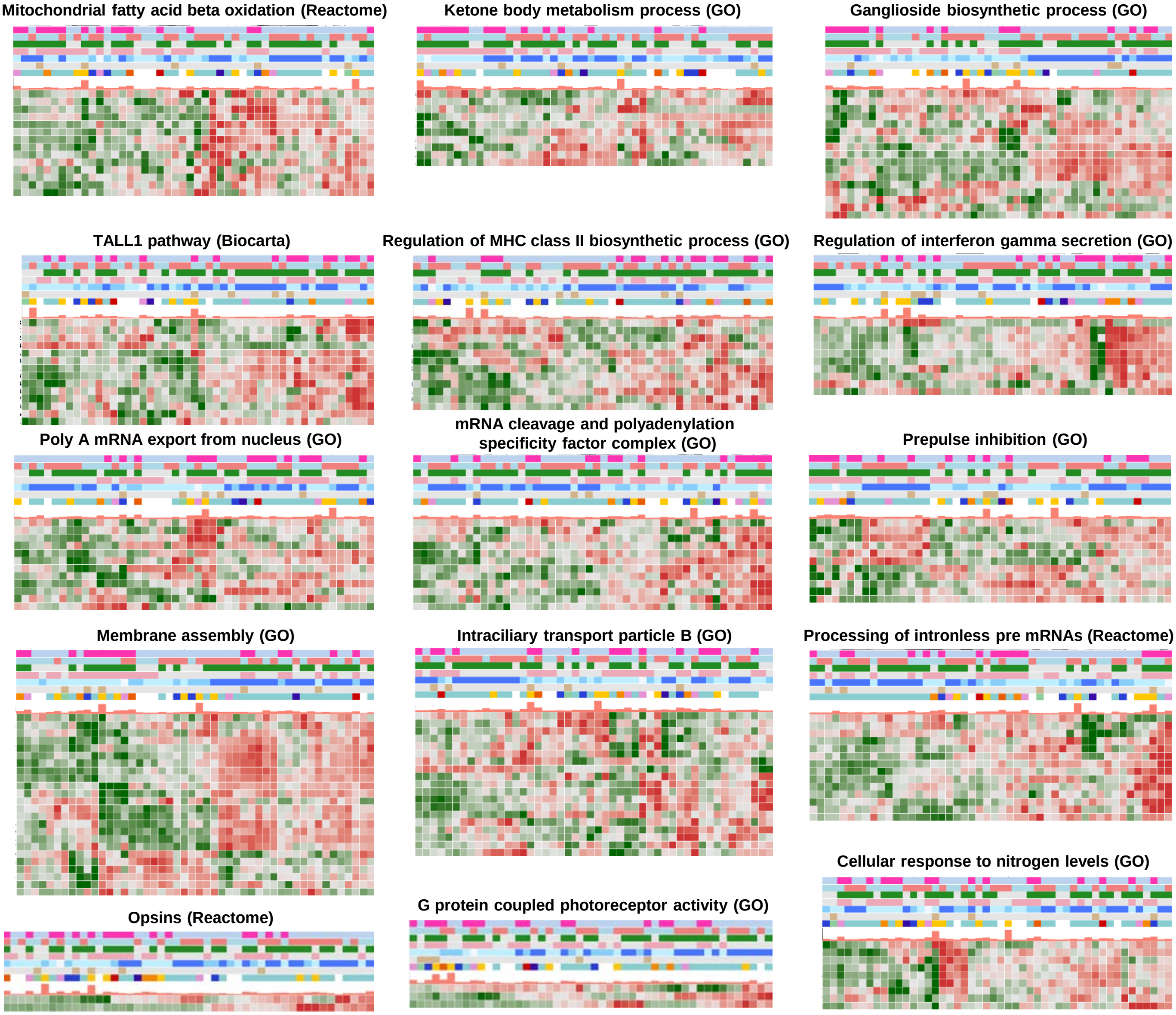

Figure S5. (C) The canonical pathways (n = 15) that were significantly associated with risk groups were obtained from REACTOME, BIOCARTA, KEGG and GO databases. None of pathways were significant after adjusting *P*-values with FDR. (D) Gene expression profiles of the pathways showing significant association to risk groups.

(E)

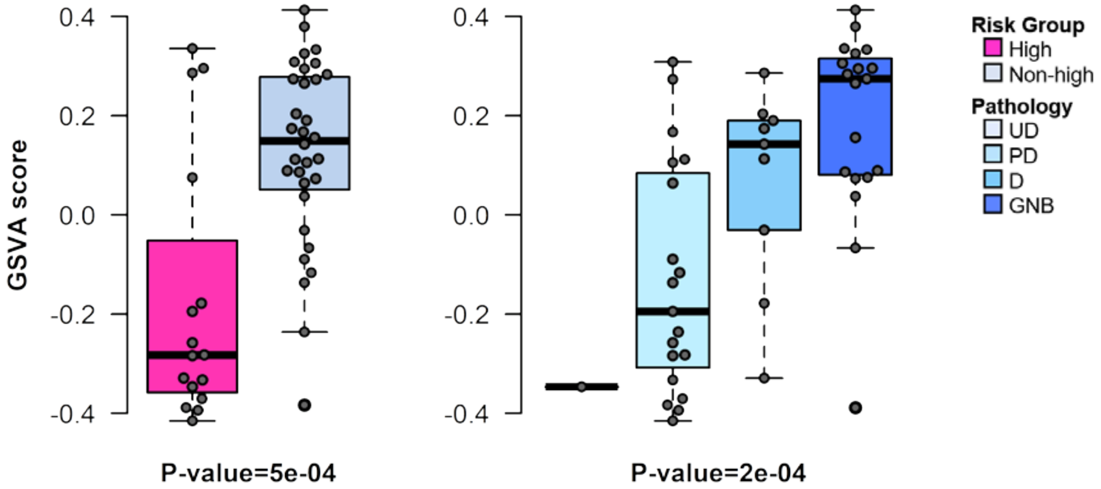

Figure S5. (E) The association of GSVA score of ganglioside biosynthesis pathway with risk-group (left) and pathology (right).
