# Supplementary material for: Genomic profile of MYCN non-amplified neuroblastoma and potential for immunotherapeutic strategies in neuroblastoma
Source: BMC Med Genomics. 2020 Nov 10;13:171. doi: 10.1186/s12920-020-00819-5 (PMC7653769; doi:10.1186/s12920-020-00819-5)
Supplement: Supplementary file 6 — Additional file 6. Result of Mutational signature analysis. [file 12920_2020_819_MOESM6_ESM.pdf]

(A)

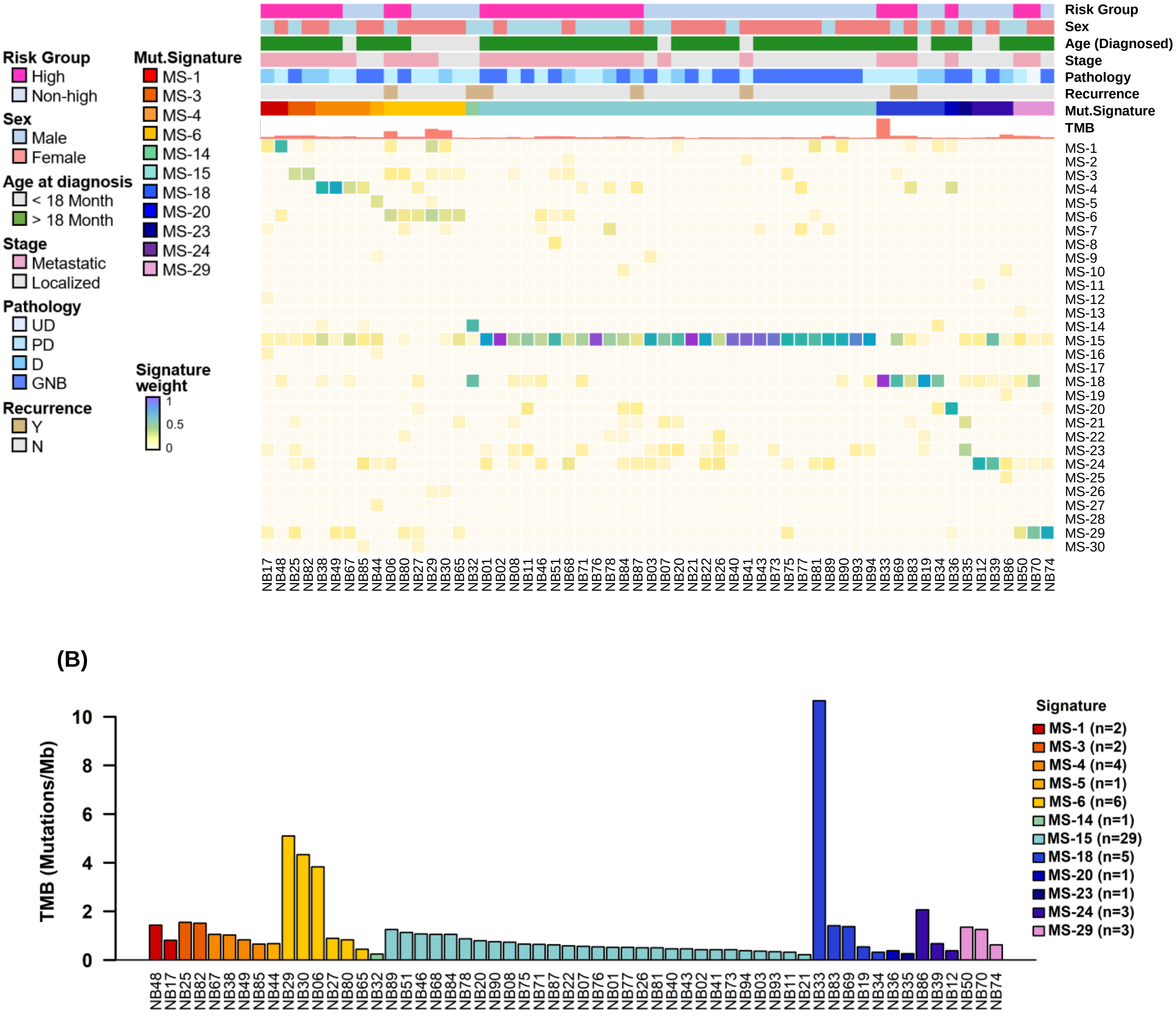

Figure S6. (A) The overview of mutational signature. The heatmap represents the signature weights predicted by deconstructSigs. Each sample was allocated to the most predominant signature out of 30 signatures. (B) Distribution of TMB grouped by mutational signature. The samples are sorted by TMB within each signature cluster.
